# *rac*-(1*R*,2*R*,4*S*)-1,2-Dibromo-4-[(1*R*)-1,2-dibromo­eth­yl]cyclo­hexa­ne

**DOI:** 10.1107/S160053681004763X

**Published:** 2010-11-27

**Authors:** Robert Köppen, Matthias Koch, Franziska Emmerling, Irene Nehls

**Affiliations:** a BAM Federal Institute for Materials Research and Testing, Department Analytical Chemistry, Reference Materials, Richard-Willstätter-Strasse 11, D-12489 Berlin-Adlershof, Germany

## Abstract

In the title compound, C_8_H_12_Br_4_, the cyclo­hexane ring exhibits a chair conformation. The C—Br distances range from 1.964 (6) to 1.985 (5) Å and the C—C distances range from 1.496 (6) to 1.543 (7) Å. Short inter­molecular Br⋯Br contacts [3.467 (4) Å] occur in the crystal.

## Related literature

The title compound is an environmentally novel brominated flame retardant (Arsenault *et al.*, 2008 ▶; de Wit *et al.*, 2010 ▶), also known as TBECH, which was recently identified in beluga whales and in the eggs of herring gulls and double-crested cormorants (Tomy *et al.*, 2008 ▶; Gauthier *et al.*, 2009 ▶). There is relatively little information available concerning the persistence of TBECH in environmental media, its bioaccumulation in food webs and the toxicity of the pure stereoisomers (Rattfelt *et al.*, 2006 ▶; Muir *et al.*, 2007 ▶; Khalaf *et al.*, 2009 ▶; Nyholm *et al.*, 2009 ▶, 2010 ▶). The Br⋯Br contacts in the crystal structure can be classified according to Ramasubbu *et al.* (1986 ▶).
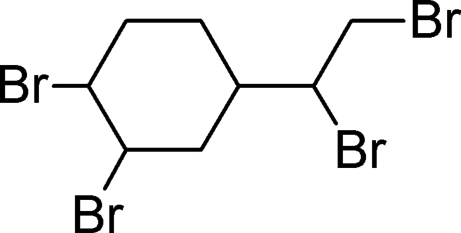


## Experimental

### 

#### Crystal data

C_8_H_12_Br_4_
*M* *_r_* = 427.82
Monoclinic, 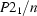

*a* = 9.6163 (14) Å
*b* = 13.9193 (19) Å
*c* = 9.6354 (15) Å
β = 111.769 (9)°
*V* = 1197.7 (3) Å^3^
*Z* = 4
Mo *K*α radiation
μ = 13.39 mm^−1^
*T* = 294 K
0.14 × 0.11 × 0.05 mm

#### Data collection

Bruker APEX CCD area-detector diffractometer
Absorption correction: multi-scan (*SADABS*; Bruker, 2001 ▶) *T*_min_ = 0.61, *T*_max_ = 0.72
20037 measured reflections
2213 independent reflections
1471 reflections with *I* > 2σ(*I*)
*R*_int_ = 0.104

#### Refinement

*R*[*F* ^2^ > 2σ(*F* ^2^)] = 0.032
*wR*(*F* ^2^) = 0.086
*S* = 1.01
2213 reflections
109 parameters
H-atom parameters constrained
Δρ_max_ = 0.74 e Å^−3^
Δρ_min_ = −0.55 e Å^−3^

### 

Data collection: *SMART* (Bruker, 2001 ▶); cell refinement: *SAINT* (Bruker, 2001 ▶); data reduction: *SAINT*; program(s) used to solve structure: *SHELXS97* (Sheldrick, 2008 ▶); program(s) used to refine structure: *SHELXL97* (Sheldrick, 2008 ▶); molecular graphics: *SHELXTL* (Sheldrick, 2008 ▶) and *ORTEPIII* (Burnett & Johnson, 1996 ▶); software used to prepare material for publication: *SHELXTL*.

## Supplementary Material

Crystal structure: contains datablocks I, global. DOI: 10.1107/S160053681004763X/sj5056sup1.cif

Structure factors: contains datablocks I. DOI: 10.1107/S160053681004763X/sj5056Isup2.hkl

Additional supplementary materials:  crystallographic information; 3D view; checkCIF report

## Figures and Tables

**Fig. 1.**
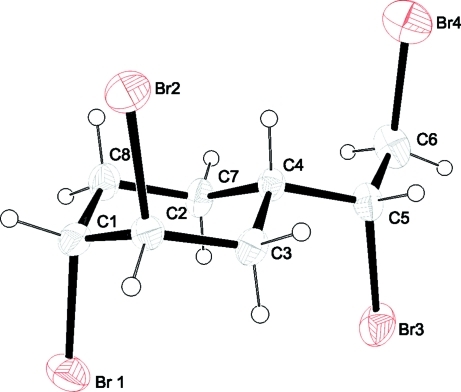
ORTEP representation of the title compound with atomic labeling shown with 30% probability displacement ellipsoids.

**Fig. 2.**
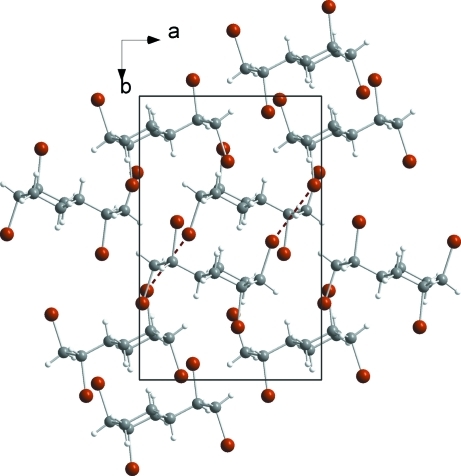
View of the unit cell of the title compound along [001] (Br: red spheres, C: gray spheres, H: light gray spheres). Short Br···Br contacts are drawn as dashed red lines.

## supplementary crystallographic information

### Comment

1,2-Dibromo-4-(1,2-dibromoethyl)cyclohexane, also known as tetrabromoethylcyclohexane (TBECH), is a cycloaliphatic brominated flame retardant used as an additive to flammable materials (*e.g.*, polystyrene and polyurethane) to decrease the risk of accidental fire (Arsenault *et al.* 2008, Tomy *et al.* 2008, de Wit *et al.* 2010). Due to the presence of 4 chiral carbons (C1, C2, C4, C5) TBECH can exist as four diastereomeric pairs of enantiomers (Arsenault *et al.* 2008). The structural differences between these stereoisomers lead to concomitant variability in physicochemical properties such as hydrophobicity and water solubility, resulting in variable propensities for biological uptake and metabolism. In this respect, the complex stereoisomerism of TBECH is a challenge for its trace quantification in relevant environmental matrices and in the food chain. Recently TBECH has been found to bioaccumulate in fish after dietary exposure (Rattfelt *et al.* 2006, Nyholm *et al.* 2009) and it was identified as a possible persistent, bioaccumulative and endocrine disrupting organohalogen chemical (Muir *et al.* 2007, Khalaf *et al.* 2009). Calculated half-lives of technical TBECH in activated aerobic and anaerobic soil at 20 °C were estimated to be 21 and 23 days, respectively (Nyholm *et al.* 2010). In the same study much slower degradation was observed during incubation at 8 °C (half-life: 120 days), suggesting that TBECH will persist in temperate climate zones for an extended period. However, the findings of TBECH in a maritime species (beluga whale) (Tomy *et al.* 2008) as well as seabirds (herring gulls and double-crested cormorants) (Gauthier *et al.* 2009) were reported for the first time.

The molecular structure of the compound and the atom-labeling scheme are shown in Fig 1. The compound crystallises as a racemate. and each molecule is involved in two intermolecular Br···Br contacts [d(Br1-Br2): 3.467 (4)Å] below the sum of their van der Waals radii, which influence the molecular packing and lead to the formation of chains along the *b* axis. Generally, halogen···halogen contacts C—*X*···*X*—C are defined as type I if the C—*X*···*X* angle θ1 is equal or nearly equal to the *X*···*X*—C angle θ2. If σimeq 180° and σimeq 90°, the contact is defined as type II (Ramasubbu *et al.* 1986). For the title compound the respective values amount to θ1(C1—Br1···Br2) = 161.2 (2)° and θ1(C2—Br2···Br1) = 137.3 (2)° These values are in acoordance with type I contacts arise as a result of close packing about an inversion center.

### Experimental

In a 2 *L* two-necked round bottom flask equipped with a thermometer and a 50 ml dropping funnel, 20.1 g (186 mmol) 4-vinylcyclohexene were dissolved in 1000 mL of dichloromethane. Bromine (19.5 ml, 381 mmol) was slowly added through the dropping funnel within 60 min. Light was excluded from the flask and the reaction mixture was stirred for 20 hrs at ambient temperature. Then excess bromine and dichloromethane were removed by rotary evaporation and the white residue was recrystallied from methanol. For single-crystal *x*-ray crystallography colourless crystals of the title compound were grown by slow solvent evaporation from methanol at ambient temperature in the absence of light.

### Refinement

The C—H hydrogen atoms were located in difference maps and and fixed in their found positions with *U*_iso_(H) = 1.2 of the parent atom *U*_eq_ or 1.5 *U*_eq_(C_methyl_).

### Crystal data

**Table:**

C_8_H_12_Br_4_	*F*(000) = 800
*M**_r_* = 427.82	*D*_x_ = 2.372 Mg m^−^^3^
Monoclinic, *P*2_1_/*n*	Mo *K*α radiation, λ = 0.71073 Å
Hall symbol: -P 2yn	Cell parameters from 48 reflections
*a* = 9.6163 (14) Å	θ = 2.2–35°
*b* = 13.9193 (19) Å	µ = 13.39 mm^−^^1^
*c* = 9.6354 (15) Å	*T* = 294 K
β = 111.769 (9)°	Block, colourless
*V* = 1197.7 (3) Å^3^	0.14 × 0.11 × 0.05 mm
*Z* = 4	

### Data collection

**Table:**

Bruker APEX CCD area-detector diffractometer	2213 independent reflections
Radiation source: fine-focus sealed tube	1471 reflections with *I* > 2σ(*I*)
graphite	*R*_int_ = 0.104
ω/2θ scans	θ_max_ = 25.4°, θ_min_ = 2.6°
Absorption correction: multi-scan (*SADABS*; Bruker, 2001)	*h* = −11→11
*T*_min_ = 0.61, *T*_max_ = 0.72	*k* = −16→16
20037 measured reflections	*l* = −11→11

### Refinement

**Table:**

Refinement on *F*^2^	Primary atom site location: structure-invariant direct methods
Least-squares matrix: full	Secondary atom site location: difference Fourier map
*R*[*F*^2^ > 2σ(*F*^2^)] = 0.032	Hydrogen site location: inferred from neighbouring sites
*wR*(*F*^2^) = 0.086	H-atom parameters constrained
*S* = 1.01	*w* = 1/[σ^2^(*F*_o_^2^) + (0.0411*P*)^2^] where *P* = (*F*_o_^2^ + 2*F*_c_^2^)/3
2213 reflections	(Δ/σ)_max_ < 0.001
109 parameters	Δρ_max_ = 0.74 e Å^−^^3^
0 restraints	Δρ_min_ = −0.55 e Å^−^^3^

### Special details

**Table:**

Geometry. All e.s.d.'s (except the e.s.d. in the dihedral angle between two l.s. planes) are estimated using the full covariance matrix. The cell e.s.d.'s are taken into account individually in the estimation of e.s.d.'s in distances, angles and torsion angles; correlations between e.s.d.'s in cell parameters are only used when they are defined by crystal symmetry. An approximate (isotropic) treatment of cell e.s.d.'s is used for estimating e.s.d.'s involving l.s. planes.
Refinement. Refinement of *F*^2^ against ALL reflections. The weighted *R*-factor *wR* and goodness of fit *S* are based on *F*^2^, conventional *R*-factors *R* are based on *F*, with *F* set to zero for negative *F*^2^. The threshold expression of *F*^2^ > σ(*F*^2^) is used only for calculating *R*-factors(gt) *etc*. and is not relevant to the choice of reflections for refinement. *R*-factors based on *F*^2^ are statistically about twice as large as those based on *F*, and *R*- factors based on ALL data will be even larger.

### Fractional atomic coordinates and isotropic or equivalent isotropic displacement parameters (Å^2^)

**Table:**

	*x*	*y*	*z*	*U*_iso_*/*U*_eq_	
Br1	−0.22923 (7)	0.01287 (4)	0.19367 (7)	0.0611 (2)	
Br2	−0.03998 (7)	0.31412 (4)	0.35632 (7)	0.0567 (2)	
Br3	0.29332 (7)	−0.05633 (4)	0.35758 (7)	0.0640 (2)	
Br4	0.48396 (8)	0.23052 (5)	0.26547 (9)	0.0742 (2)	
C1	−0.1618 (6)	0.1472 (3)	0.1927 (6)	0.0424 (13)	
H1	−0.2493	0.1896	0.1612	0.051*	
C2	−0.0616 (5)	0.1722 (3)	0.3510 (6)	0.0366 (12)	
H2	−0.1122	0.1535	0.4184	0.044*	
C3	0.0879 (5)	0.1238 (3)	0.4013 (5)	0.0377 (12)	
H3A	0.0747	0.0558	0.4150	0.045*	
H3B	0.1510	0.1501	0.4972	0.045*	
C4	0.1672 (5)	0.1358 (3)	0.2906 (5)	0.0340 (12)	
H4	0.1874	0.2045	0.2864	0.041*	
C5	0.3200 (6)	0.0841 (4)	0.3478 (6)	0.0436 (13)	
H5	0.3768	0.1071	0.4492	0.052*	
C6	0.4170 (6)	0.0966 (4)	0.2563 (7)	0.0596 (16)	
H6A	0.3606	0.0789	0.1532	0.072*	
H6B	0.5033	0.0545	0.2945	0.072*	
C7	0.0666 (5)	0.1057 (4)	0.1343 (5)	0.0434 (13)	
H7A	0.1158	0.1201	0.0652	0.052*	
H7B	0.0502	0.0369	0.1325	0.052*	
C8	−0.0828 (6)	0.1569 (4)	0.0840 (6)	0.0450 (14)	
H8A	−0.0671	0.2245	0.0705	0.054*	
H8B	−0.1469	0.1312	−0.0120	0.054*	

### Atomic displacement parameters (Å^2^)

**Table:**

	*U*^11^	*U*^22^	*U*^33^	*U*^12^	*U*^13^	*U*^23^
Br1	0.0576 (4)	0.0503 (3)	0.0783 (5)	−0.0152 (3)	0.0285 (4)	−0.0085 (3)
Br2	0.0605 (4)	0.0369 (3)	0.0721 (5)	0.0061 (3)	0.0240 (4)	−0.0052 (3)
Br3	0.0600 (4)	0.0451 (3)	0.0740 (5)	0.0157 (3)	0.0100 (4)	−0.0049 (3)
Br4	0.0771 (5)	0.0802 (5)	0.0861 (6)	−0.0076 (4)	0.0545 (4)	−0.0118 (4)
C1	0.040 (3)	0.038 (3)	0.050 (4)	0.006 (2)	0.018 (3)	0.007 (2)
C2	0.036 (3)	0.037 (3)	0.040 (3)	0.003 (2)	0.018 (3)	0.001 (2)
C3	0.041 (3)	0.043 (3)	0.025 (3)	0.005 (2)	0.008 (3)	−0.001 (2)
C4	0.032 (3)	0.035 (3)	0.030 (3)	0.001 (2)	0.006 (2)	−0.004 (2)
C5	0.035 (3)	0.047 (3)	0.045 (4)	0.003 (2)	0.010 (3)	−0.011 (3)
C6	0.048 (4)	0.067 (4)	0.066 (4)	0.001 (3)	0.025 (3)	−0.023 (3)
C7	0.036 (3)	0.061 (3)	0.032 (3)	0.011 (3)	0.011 (3)	0.003 (3)
C8	0.042 (3)	0.056 (3)	0.034 (3)	0.004 (3)	0.010 (3)	0.005 (3)

### Geometric parameters (Å, °)

**Table:**

Br1—C1	1.980 (5)	C4—C7	1.516 (7)
Br2—C2	1.985 (5)	C4—C5	1.543 (7)
Br3—C5	1.979 (5)	C4—H4	0.9800
Br4—C6	1.964 (6)	C5—C6	1.512 (7)
C1—C8	1.511 (7)	C5—H5	0.9800
C1—C2	1.512 (7)	C6—H6A	0.9700
C1—H1	0.9800	C6—H6B	0.9700
C2—C3	1.496 (6)	C7—C8	1.513 (7)
C2—H2	0.9800	C7—H7A	0.9700
C3—C4	1.533 (7)	C7—H7B	0.9700
C3—H3A	0.9700	C8—H8A	0.9700
C3—H3B	0.9700	C8—H8B	0.9700
			
C8—C1—C2	112.5 (4)	C6—C5—C4	116.8 (5)
C8—C1—Br1	109.7 (3)	C6—C5—Br3	105.1 (3)
C2—C1—Br1	107.5 (3)	C4—C5—Br3	110.8 (3)
C8—C1—H1	109.0	C6—C5—H5	108.0
C2—C1—H1	109.0	C4—C5—H5	108.0
Br1—C1—H1	109.0	Br3—C5—H5	108.0
C3—C2—C1	113.5 (4)	C5—C6—Br4	110.2 (4)
C3—C2—Br2	111.2 (3)	C5—C6—H6A	109.6
C1—C2—Br2	106.1 (3)	Br4—C6—H6A	109.6
C3—C2—H2	108.7	C5—C6—H6B	109.6
C1—C2—H2	108.7	Br4—C6—H6B	109.6
Br2—C2—H2	108.7	H6A—C6—H6B	108.1
C2—C3—C4	113.1 (4)	C8—C7—C4	111.6 (4)
C2—C3—H3A	109.0	C8—C7—H7A	109.3
C4—C3—H3A	109.0	C4—C7—H7A	109.3
C2—C3—H3B	109.0	C8—C7—H7B	109.3
C4—C3—H3B	109.0	C4—C7—H7B	109.3
H3A—C3—H3B	107.8	H7A—C7—H7B	108.0
C7—C4—C3	111.2 (4)	C1—C8—C7	113.4 (4)
C7—C4—C5	113.5 (4)	C1—C8—H8A	108.9
C3—C4—C5	110.8 (4)	C7—C8—H8A	108.9
C7—C4—H4	107.0	C1—C8—H8B	108.9
C3—C4—H4	107.0	C7—C8—H8B	108.9
C5—C4—H4	107.0	H8A—C8—H8B	107.7
			
C8—C1—C2—C3	−48.8 (6)	C7—C4—C5—Br3	−61.2 (5)
Br1—C1—C2—C3	72.1 (4)	C3—C4—C5—Br3	64.7 (4)
C8—C1—C2—Br2	73.5 (4)	C4—C5—C6—Br4	66.9 (5)
Br1—C1—C2—Br2	−165.6 (2)	Br3—C5—C6—Br4	−170.0 (3)
C1—C2—C3—C4	50.4 (6)	C3—C4—C7—C8	53.3 (5)
Br2—C2—C3—C4	−69.1 (5)	C5—C4—C7—C8	179.0 (4)
C2—C3—C4—C7	−52.6 (5)	C2—C1—C8—C7	50.4 (6)
C2—C3—C4—C5	−179.7 (4)	Br1—C1—C8—C7	−69.2 (5)
C7—C4—C5—C6	59.0 (6)	C4—C7—C8—C1	−53.2 (6)
C3—C4—C5—C6	−175.1 (4)		
